# Revealing the Characteristics and Correlations Among Microbial Communities, Functional Genes, and Vital Metabolites Through Metagenomics in Henan Mung Bean Sour

**DOI:** 10.3390/microorganisms13040845

**Published:** 2025-04-07

**Authors:** Xunda Wang, Yue Li, Lei Zuo, Pengna Li, Haiwei Lou, Renyong Zhao

**Affiliations:** 1 College of Food Science and Engineering, Henan University of Technology, Lianhua Street, Hi-Tech Development Zone, Zhengzhou 450001, China15729397701@163.com (Y.L.); zl2413324506@163.com (L.Z.); 18317406931@163.com (P.L.); superharry@163.com (H.L.); 2 Food Laboratory of Zhongyuan, Luohe 462300, China

**Keywords:** Henan mung bean sour, metagenomics, dominant genera, metabolites, functional genes

## Abstract

Henan mung bean sour (HMBS) is the raw material for mung bean sour noodles (MBSNs), a traditional fermented food. To investigate the characteristic flavor compounds, we have detected the content of free amino acids (FAAs) and key metabolites including organic acids, sugars, and alcohols. The results revealed that the content associated with umami, sweetness, and bitterness (TVA > 1) showed significant differences. Metagenomic analysis indicated that *Lactobacillus delbrueckii* was the dominant and characteristic species in WJ and LY15, whereas *Bifidobacterium mongoliense*, *Lactiplantibacillus plantarum*, and *Acetobacter indonesiensis* were the dominant species in GY. The abundance of functional genes related to carbohydrate and amino acid metabolism was higher in WJ and LY15. There was a strong correlation between dominant genera and vital metabolites (r |>| 0.7). This study provides a theoretical foundation for the development of HMBS.

## 1. Introduction

Henan mung bean sour (HMBS) is a traditional delicacy popular in the Central Plains region of China, particularly in Henan Province. The region is home to nearly 100 million people. After cooking, HMBS releases a pleasant sour and savory taste, and when paired with ingredients like noodles and celery, it forms a dish known as “mung bean sour noodles”, which is highly regarded by locals [1,2]. Studies have shown that natural fermentation can enhance the nutritional density of mung beans. Microorganisms such as lactic acid bacteria and yeasts break down the large molecules in mung beans, converting them into smaller, more digestible components, which significantly increases the protein and amino acid content [3,4]. Additionally, fermentation partially hydrolyzes the starch in mung beans, reducing their beany odor and improving taste [5,6]. Functional compounds in mung beans, such as polyphenols, amino acids, and Gamma-aminobutyric acid, also increase or are generated during the fermentation process [7,8]. However, there is a lack of research on the content of key metabolites, such as organic acids and free amino acids, in different varieties of HMBS after natural fermentation. In the fermentation process, microorganisms such as bacteria, yeasts, and molds contribute to the development of flavor, texture, and nutritional properties of food [4,9]. Compared to 16S rRNA sequencing, metagenomics offers advantages including higher taxonomic resolution, the capability for functional gene analysis, detection of low-abundance microorganisms, broader coverage of microbial taxa, and avoidance of PCR amplification bias. In fermented dairy products, such as yogurt and cheese, metagenomics has been used to study the microbial diversity and metabolic pathways that contribute to texture, aroma, and taste [10,11]. Metagenomics enables direct detection of all genes within a sample—including functional genes, metabolic pathways, antibiotic resistance genes, and others—providing a comprehensive analysis of the functional potential of microbial communities without reliance on predictive methods. This is critical for elucidating the mechanisms driving HMBS flavor formation. For example, Wang et al. [2] used traditional high-throughput sequencing and were only able to identify microbial communities at the genus level [2]. They found that the HMBS samples prepared by dry methods were predominantly composed of *Lactobacillus* and *Pediococcus*. This limitation severely restricts further research. Traditional isolation and screening methods also have significant limitations for comprehensively claiming the members in the microbial community [12]. Therefore, it is essential to apply metagenomics to deeply investigate the composition of the microbial community and their functional genes in HMBS.

Amino acids play a crucial role in both the flavor formation and nutritional enhancement of fermented foods. Microorganisms transform amino acids into a variety of flavor-active compounds, which significantly influence the taste, aroma, and overall flavor profile of fermented products, such as esters, aldehydes, and ketones [13]. These compounds contribute rich and complex flavors to foods. In particular, the conversion of amino acids directly affects the final taste of fermented foods like soy-based products, dairy, and meats [9,14]. Additionally, amino acids participate in the Maillard reaction, a key chemical reaction that generates aromatic compounds in fermented foods like bread, beer, and cheese, contributing to the development of complex aromas and flavors [11]. Amino acids serve as critical taste-active compounds, with glutamic acid (Glu) and aspartic acid (Asp) representing canonical umami-enhancing substances. Notably, bitter-taste amino acids such as valine (Val) and lysine (Lys) exhibit significant synergistic interactions with umami-associated amino acids in flavor modulation [15]. During lactic fermentation, organic acids, such as acetic acid and propionic acid, confer a unique sour taste to dairy products. Similarly, organic acids like lactic acid, citric acid, and formic acid play indispensable roles in the flavor development and preservation of fermented foods [5,16]. However, those previous studies relating free amino acids and organic acids in different HMBS are absent, and the role of microorganisms in driving the amino acid and carbohydrate metabolism within HMBS remains unclear. Polyphenolic compounds exhibit bioactive properties including antioxidant, anti-inflammatory, and anticancer effects [17]. Microbial fermentation has been shown to enhance these biological activities. However, limited research has been conducted on the transformation of polyphenolic flavonoids during mung bean fermentation [18,19].

Therefore, to investigate whether microorganisms in HMBS possess functional genes capable of converting and amplifying polyphenolic bioactivity, metagenomic analysis emerges as an essential methodological approach. This study aims to comprehensively elucidate the composition of microbial communities and functional genes in various HMBS samples through metagenomics. The free amino acid and organic acid content will be measured and Pearson’s correlation coefficient will be used to explore the potential relationship between these compounds and the dominant genera. This research will lay the foundation for the further standardization and industrial development of HMBS products.

## 2. Materials and Methods

### 2.1. Chemicals and Reagents

Ultrapure water was purchased from Vazyme Corp. (Nanjing, China). The sulfuric acid was LC-MS grade and purchased from Merck (Darmstadt, Germany). The formic acid, lactic acid, glucose, fructose, acetic acid, citric acid, and ethanol were obtained from Aladdin (Shanghai, China). Standards of 17 free amino acids were purchased from Merck (Germany).

### 2.2. Sample Collection

Five-liter samples were purchased from Dunzhi Street 15 in Luoyang, Wangji Workshop in Luoyang, and Gongyi County in Zhengzhou and were labeled as LY15, WJ, and GY, respectively. The samples were immediately stored in a portable freezer at 4 °C and centrifuged within 6 h into 15 mL centrifuge tubes. These were stored at 4 °C, −18 °C, and −80 °C for subsequent metagenomic sequencing, physico-chemical analysis, and sensory evaluation, respectively.

### 2.3. Sensory Evaluation of Henan Mung Bean Sour

We followed the traditional preparation method by mixing HMBS with boiling water at a 1:3 ratio, then boiling it for 5 min and cooling it to room temperature. We recruited 12 sensory evaluators (aged 20–30, with a gender ratio of 1:1). They were trained based on prior research to enable them to make independent judgments [20]. Cooked samples were marked with random letters. The panels accepted three samples of every type of HMBS in a plastic cup (5 mL) on a clean plate. The samples were tested at 30 °C. Following a sensory evaluation test, we measured the color, smell, texture, mouth feel, and taste. Each criterion has a maximum score of 25, with a total maximum score of 100.

### 2.4. Total Titrable Acid and pH Measurement

To determine pH of each sample, a sample (15 mL) was added to distilled water (85 mL) [21]. The pH of the treated samples was measured at 15 min. To measure the TTA of HMBS, the titration method of AOAC (1990) was performed by the pH meter (Mettler Toledo, Greifensee, Switzerland). A HMBS sample (10 mL) was mixed with 90 mL of distilled water. We dropped phenolphthalein into the solution (20 mL) and then consumed it using 0.1 N NaOH. The TTA was recorded with the following formula:

TTA (%) = (V × F × 0.009/m) × 100,

where V means the volume of 0.1 N NaOH (mL), F represents the factor of 0.1 N NaOH (mL), m represents the weight of the sample, and 0.009 means the lactic acid amount equivalent to 1 mL of 0.1 N NaOH.

### 2.5. Determination of Free Amino Acids and Vital Metabolites

The method for determining free amino acids follows our previous studies [2]. The experiment was performed from the standard of GB5009.124-2016 [22]. The vital metabolites were measured by the previous method [2].

### 2.6. Microbial Community and Functional Annotation by Metagenomics

#### 2.6.1. DNA Extraction and Metagenomic Sequencing

Mung bean sour samples were collected and kept in sterile plastic bags during transport to the laboratory. The organisms were washed twice with pre-cooled phosphate buffered saline solution, centrifuged at 4000× *g* for 3 min at 4 °C, the supernatant was removed, and the bottom organism precipitate was retained, snap-frozen in liquid nitrogen, and stored at −80 °C. Samples were stored in a refrigerator −80 °C. Total DNA was extracted by Genomic DNA Preparation Kit (G2N350, Sigma, Cream Ridge, NJ, USA). The concentration and purity of the DNA were detected by ScanDrop 250 (Jena, Germany). The concentration of metagenomic DNA was eligible at 10–50 ng/μL. DNA purity and integrity were detected by 1% agarose gel electrophoresis. The electrophoresis was carried out at 90 V for 30 min. DNA fragmentation was performed to create 350 bp fragments (Covaris M220). A paired-end (PE) library was constructed with “Y”-shaped adapters attached. Magnetic bead selection was used to remove self-ligated adapter fragments, followed by PCR amplification to enrich the library template. Denaturation with sodium hydroxide generated single-stranded DNA fragments. Bridge PCR, one end of the DNA fragment, complementary to the primer bases, was anchored on the chip. The other end was randomly paired with a nearby primer, also becoming fixed, forming a “bridge” for PCR amplification. This process produced DNA clusters, which were then linearized into single-stranded DNA amplicons. According to the standard operating procedures of the Illumina platform, sequencing libraries and metagenomic data analysis were constructed. The metagenomic sequencing were carried out by Majorbio corporation (Shanghai, China) [16].

#### 2.6.2. Bioinformatics Analysis

According to the previous method [14], data analysis begins with the raw sequences from sequencing, which undergo optimization steps such as splitting, quality trimming, and contaminant removal. The optimized sequences are then used for assembly and gene prediction. The identified genes are annotated and classified by species and function, including databases like NR, COG, CAzy, and KEGG. Using DIAMOND software (Version 1.0) (http://ab.inf.unituebingen.de/software/diamond/ (accessed on 6 September 2024)), the non-redundant (NRA) gene set was aligned with the NR database (alignment type: BLASTP). Species annotations were then ensured through the taxonomic information in the NR database. The abundance of each species was calculated by summing their gene abundances. This abundance data was further classified at various taxonomic levels including Domain, Kingdom, Phylum, Genus, and Species. Using the scan tool from the CAZy database, the non-redundant gene set was aligned with CAZy database, with the expectation value (e-value) parameter set to 1 × 10^−5^. This alignment provided annotations for genes corresponding to carbohydrate-active enzymes. The richness of each carbohydrate-active enzyme was then calculated by summing the abundances of the genes referred with it. Metabolic pathways and enzymes involved in the vital metabolites of HMBS were identified using the KEGG database and previous studies [23].

The software and database of bioinformatics analysis were followed, using the NR (Non-Redundant) database, (Version 20200604, https://ftp.ncbi.nlm.nih.gov/blast/ (accessed on 16 September 2024)), KEGG (Version 94.2, www.genome.jp/kegg (accessed on 20 September 2024)), CAzy (Version 8, www.cazy.org (accessed on 6 September 2024)), and Python (Version 2.7, Version 3.5), R (Version 3.3.1, Version 3.3.3).

### 2.7. Statistical Analysis

All analyses were performed on at least three replicates per sample. Results were showed as the mean value ± standard deviation (mean ± SD, *n* = 3). By IBM SPSS Statistics (version 25.0, Armonk, NY, USA), one-way analysis of variance (ANOVA) followed by Duncan’s multiple range test was conducted. SIMCA software (version 14.1, Umeå, Sweden) was employed to carry the partial least squares discriminant analysis (PLS-DA) to detect the differences in free amino acids among HMBS samples. The Pearson’s correlation index was calculated by Origin (version 2023). GraphPad Prism (version 7, Boston, MA, USA) was used for data plotting.

## 3. Results and Discussion

### 3.1. Sensory Evaluation and pH Changes of Different HMBS

**Figure 1:**
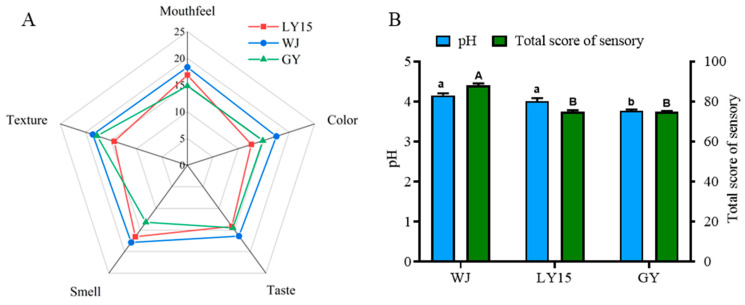
(**A**) Radar chart of sensory evaluation based on K-means clustering; (**B**) comparison of pH and total sensory scores of different samples. TVA, taste activity value. VIP, variable importance in the projection. Significant differences are denoted by different lowercase letters and uppercase letters (*p* < 0.05).

The evaluation of HMBS sensory characteristics lacked established standards, which prompted us to conduct a sensory assessment based on similar products such as yogurt [24,25]. Twelve evaluators assessed color, texture, smell, taste, and mouth feel, each scoring out of 25 points (Figure 1A). Color, a key indicator of food quality, was highest in the WJ sample (18 points), followed by GY and LY15, with the latter scoring the lowest (13 points). Similarly, WJ was superior to GY and LY15 in taste, smell, texture and so on. Notably, GY had the lowest smell score (13 points), likely due to spoilage or pathogenic bacteria [4]. LY15 had the lowest texture score (15), while WJ and GY had similar scores of 18. Texture differences are linked to starch and protein content and their structural changes [26]. These results suggest that WJ’s microbial composition fosters a balanced production of beneficial compounds, improving sensory quality. WJ also has the highest total sensory score (91), with GY and LY15 following. The pH of GY was lowest (3.80), while LY15 and WJ were similar (close to 4), with a low pH inhibiting harmful bacteria but potentially suppressing flavor compounds. Compared to the previous study by Li et al., this research newly collected GY samples for analysis, revealing a significantly lower pH level than WJ and other samples. This discrepancy could be attributed to regional climatic variations, differences in processing methods, and distinctions in domestic water usage patterns.

### 3.2. Role of Characteristic Free Amino Acids

Free amino acids (FAAs) significantly influence the flavor of fermented food by enhancing umami, sweet, sour, and bitter tastes, with glutamate, asparagine, and arginine being key contributors [4,27]. Table 1 presents concentrations, total volatile acids (TVAs), and VIP values for 17 FAAs in different HMBS samples. GY exhibits higher concentrations and TVA values of asparagine (Asp) and glutamate (Glu) than WJ and LY15, with Glu being the predominant umami amino acid across all samples, suggesting a correlation with higher globulin and glutamic acid content in mung beans [8]. Among sweet amino acids, methionine (Met) and alanine (Ala) dominate, with LY15 showing the highest TVA value for Ala (7.16), surpassing GY (5.16) and WJ (4.66). Bitter amino acids, particularly valine (Val), histidine (His), and lysine (Lys), contribute to bitterness, with distinct TVA variations across samples. His shows little variation, while Lys exhibits significant differences, highlighting sample-specific bitterness profiles. Tyr, the only astringent amino acid, has a TVA of 1.78 in GY, but lower values in WJ and LY15 (<1). Although GY exhibits higher TVA values in taste-related amino acids, its overall sensory score remains lower than WJ. This discrepancy likely arises from an imbalance in amino acid ratios and the influence of microbial community composition and metabolic pathways, phenomena also observed in other fermented foods [28,29].

**Figure 2:**
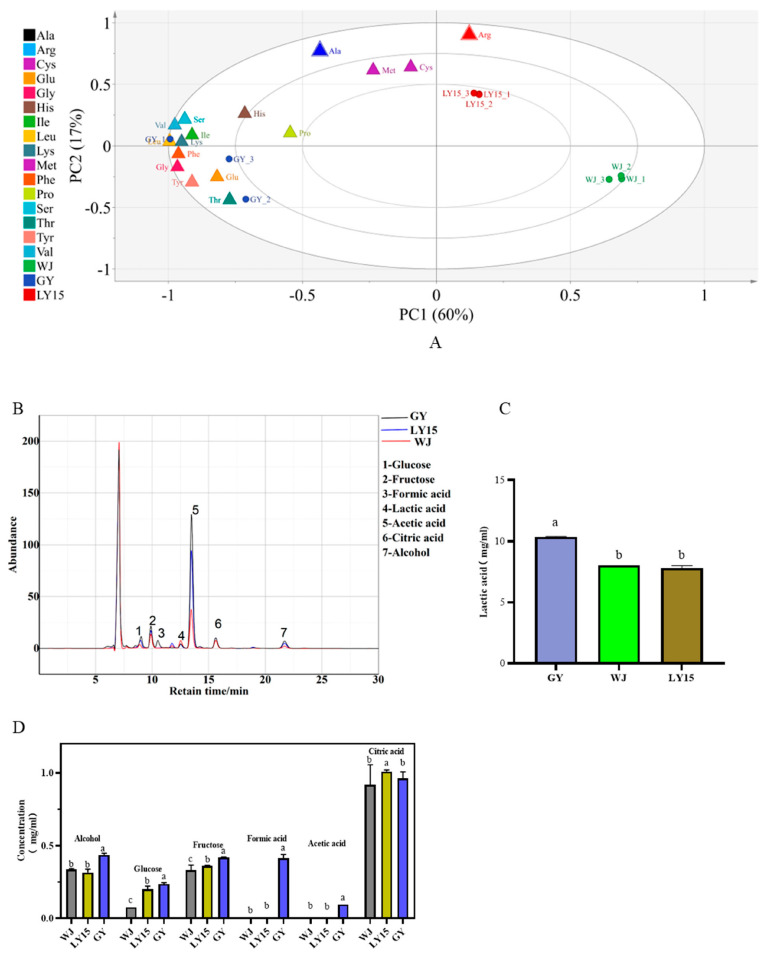
(**A**) Free amino acids profiles were subjected to partial least squares analysis (PLS-DA) R2X = 0.993, R2Y = 0.999, Q2 = 0.993. (**B**) Changes in critical metabolites in a waterfall plot in different samples. The content changes of vital metabolites in different samples (**C**,**D**). Data with different lowercases in the same index were significantly different.

PLS-DA analysis (Figure 2), with PC1 and PC2 explaining 60% and 17% of the variance, respectively, shows distinct clustering within the GY, LY15, and WJ groups, indicating both strong within-group consistency and significant inter-group differences. Glu, Lys, Val, and Ser cluster near GY, suggesting Gly and Val as characteristic FAAs of GY. Met and Arg cluster near LY15, with VIP values of 0.90 and 1.28, marking them as key FAAs for LY15. Interestingly, WJ does not cluster with any specific amino acid group, indicating a more balanced amino acid composition, which may account for its higher sensory scores.

### 3.3. Comparison of the Content of Organic Acids, Sugar, and Alcohol

In traditional fermented foods, the abundance of metabolites was the valuable clue to monitor the metabolic pathways and the metabolic intensity of the microbial community. Those typical metabolites were essential for a deeper understanding of the flavor formation and improving the quality in fermented food. By employing high-performance liquid chromatography (HPLC) in combination with the external standard method (Figure 2B), the concentrations of key metabolites were accurately compared [30]. In HMBS samples, the lactic acid content in GY was significantly higher than that in WJ and LY15 samples, reaching 1.2 mg/mL. The concentration of lactic acid in both WJ and LY15 samples also reached 8 mg/mL (Figure 2C). The ethanol concentration in the GY samples reached 0.45 mg/mL that was significantly higher than that in the WJ and LY15 samples (Figure 2D). Interestingly, the concentration of glucose in WJ was only 0.1 mg/mL that was significantly lower than that in the GY and LY15 samples. The fructose concentrations in all three samples were similar, but formic acid and acetic acid were not detected in the WJ and LY15 samples (Figure 2D). In contrast, GY samples contained formic acid and acetic acid at concentrations of 0.3 and 0.08 mg/mL, respectively. *Acetobacter* secrete acetic acid through the oxidation of ethanol and its concentration is typically kept low to reduce toxicity to the bacteria [31]. These findings suggest that GY samples may harbor a high abundance of *Acetobacter*. Formic acid, known for its antimicrobial properties, can inhibit *Salmonella* spp. and other foodborne pathogens [32]. Citric acid is an intermediate product in metabolic processes (TCA) accumulating during fermentation when the activity of aconitase and isocitrate dehydrogenase in microorganisms is low, while the activity of citrate synthetase remains high. The concentration of citric acid in the three HMBS samples was relatively high, over 0.8 mg/mL (Figure 2D), which was caused by the abundance of functional genes related to the TCA in the bacteria [33]. The composition variations of the microbial communities and functional gene differences were responsibility to the differences in organic acids, alcohols, and sugars among the three HMBS samples. Similar findings have been reported in other fermented food like kimchi and yogurt [34,35]. Employing metagenomics and other methods is indispensable to elucidate the underlying mechanism for these different.

### 3.4. Microbial Community Composition Analysis

#### 3.4.1. Change in Microbial Diversity During Fermentation

**Figure 3:**
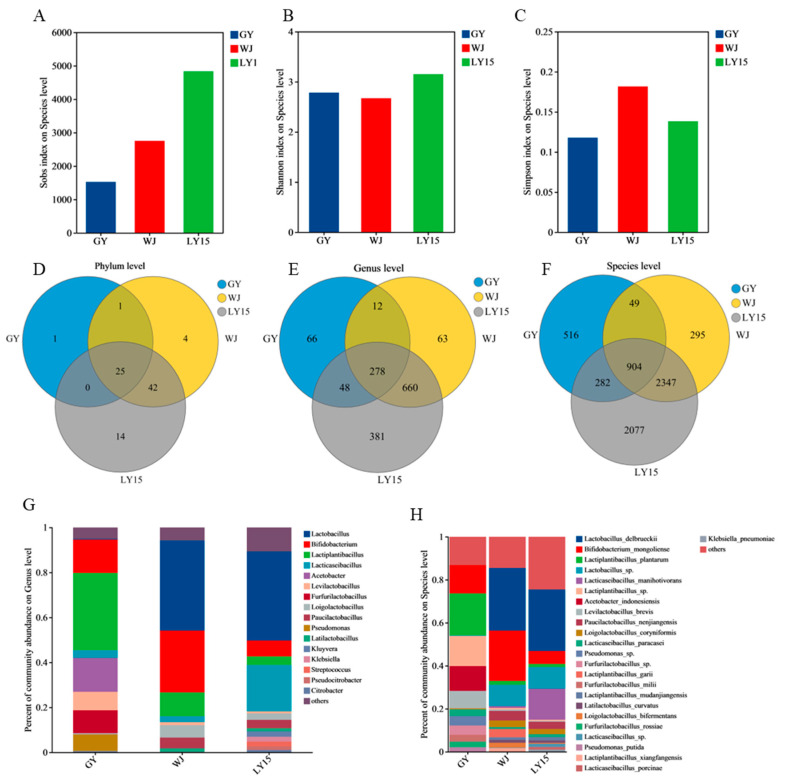
Differences in microbial community composition across samples from different regions. Variations in alpha diversity indices among samples are shown, including the Sobs index (**A**), Shannon index (**B**), and Simpson index (**C**). Venn diagrams depict phylum level (**D**), genus level (**E**), and species level (**F**), respectively. Relative abundance of microbial community at the genus (**G**) and species (**H**) level.

**Figure 4:**
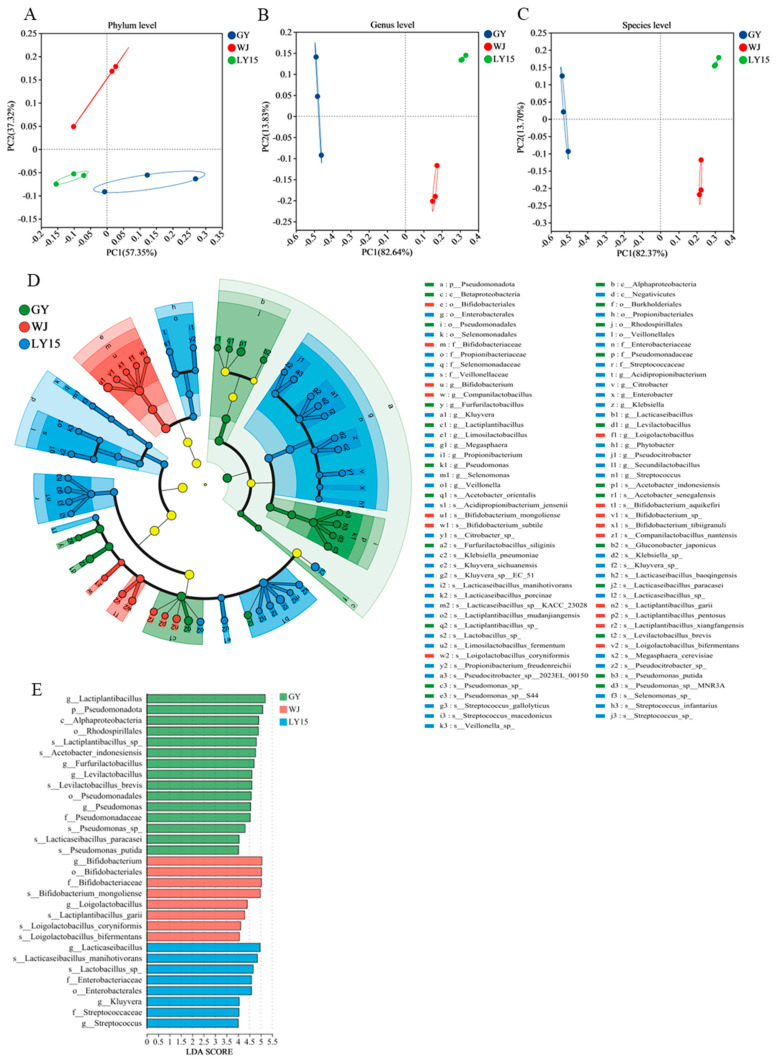
Beta diversity analysis and identification of different flora. PCoA score plots from all samples at the phylum level (**A**), genus level (**B**), and species level (**C**). Cladogram of LEfSe analysis based on shotgun metagenomics at different taxonomic levels (**D**). LEfSe differential analysis (LDA > 4) (**E**).

We have investigated the members of microbial communities of HMBS through metagenomic taxonomy annotation. The α diversity indexes, namely Sobs, Shannon, and Simpson indexes, were primarily utilized to detect the richness of microbial community on a species level (Figure 3A–C). The Sobs index was used to assess community richness, with high values indicating high richness. The community richness increased from GY, WJ to LY15 that suggested that LY15 has a larger number of bacterial species. Furthermore, the Shannon and Simpson indexes were used to estimate community diversity. A lower Simpson index means a higher community diversity. The results show that community diversity of LY15 was higher than WJ and GY. At the phylum, genus, and species levels, the number of microbial types is displayed through Venn diagrams in Figure 3D, E, and F, respectively. At the phylum level, the number of shared microbial phyla among LY15, GY, and WJ was 25, with unique phyla were at 14, 1, and 4, respectively. Similarly, the number of microbial species shared by the three groups at the genus and species levels reached 278 and 904, respectively. In contrast, the WJ sample exhibited the lowest number at both the genus and family levels, with 63 and 295 species, respectively. These findings align with the alpha diversity analysis results. Specifically, LY15 demonstrates the highest microbial richness, whereas GY exhibits a greater proportion of dominant microbial groups. The β diversity at the phylum, genus, and species levels were calculated to assess similarity among different samples through PCoA (Figure 4A–C). The results show that the GY sample was different from WJ and LY15 at the phylum level. There were significant differences in microbial composition between GY, LY15, and WJ at genus and species levels.

#### 3.4.2. The Changes of Microbial Composition in HMBS

The community compositions at the phylum, genus, and species levels for the GY, WJ, and LY15 samples are shown in Supplementary—Figure S1, Figure 3G, and Figure 3H, respectively. *Bacillota*, *Actinomycetota*, and *Pseudomonadota* were the dominant phyla across all samples, with significant differences observed at both the genus and species levels. As indicated in Table 2 and Figure 3G, the GY sample was primarily dominated by *Lactiplantibacillus* (34.40%), *Bifidobacterium* (16.38%), *Acetobacter* (15.27%), *Furfurilactobacillus* (10.29%), and *Levilactobacillus* (8.06%) at the genus level. In contrast, the LY15 and WJ samples had *Lactobacillus*, *Lacticaseibacillus*, and *Bifidobacterium* as the predominant genera, though their relative abundances varied. *Lactobacillus* constituted over 40% of the genera in both WJ and LY15, while it was underrepresented in GY. *Bifidobacterium*, known for its health benefits [36], was more abundant in WJ and GY than in LY15. Notably, *Acetobacter* and *Furfurilactobacillus* were absent in LY15 and WJ samples, likely due to the higher sensitivity and accuracy of metagenomic analysis compared to 16S rRNA sequencing [14]. Species with relative abundances greater than 1% were selected for further analysis (Figure 3H). In GY, the dominant species were mainly composed of *Bifidobacterium mongoliense*, *Lactiplantibacillus plantarum*, *Acetobacter indonesiensis*, and *Levilactobacillus brevis*. *Lactobacillus delbrueckii*, *Bifidobacterium mongoliense*, and *Lacticaseibacillus manihotivorans* in WJ and LY15 (Supplementary—Table S1). Interestingly, *Lactobacillus delbrueckii* was abundant in LY15 and WJ but scarce in GY, while *Bifidobacterium mongoliense* was most abundant in WJ (25.50%), followed by GY (14.76%) and LY15 (6.85%). As a characteristic species in GY (Supplementary—Table S1), *Acetobacter indonesiensis* was widely found in fermented vinegar and traditional Indian products [9,10].

#### 3.4.3. Screening out the Characteristic Members at Phylum, Genus, and Species Level in HMBS

The LEfSe algorithm was utilized to identify enriched features HMBS sample, considering only taxa with an average relative abundance of more than 0.5 % for the analysis (Figure 4D,E). Linear Discriminant Analysis Effect Size (LEfSe) is a bioinformatics tool designed to identify high-dimensional biomarkers and uncover genomic features (including genes, metabolic pathways, and taxonomic classifications) that differentiate between two or more biological conditions or groups. The analytical workflow consists of three key steps. The software employs a non-parametric factorial Kruskal–Wallis sum-rank test to detect features with statistically significant abundance variations across biological groups. Features showing differential abundance are subsequently evaluated using pairwise Wilcoxon rank-sum tests to verify the consistency of these differences across biological subgroups. Finally, LEfSe applies linear discriminant analysis (LDA) to estimate the magnitude of each component’s (species, gene, or functional unit) contribution to the observed differences in abundance between groups. Overall, white GY exhibited the highest number of significantly enriched taxa compared to the other two groups, followed by WJ, while LY15 showed the least enrichment (LDA, Linear Discriminant Analysis > 4). At the phylum level, *Pseudomonadota* was significantly enriched in GY sample. At the genus level, *Furfurilactobacillus*, *Levilactobacillus*, and *Pseudomonas* were significantly enriched in GY; *Bifidobacterium* and *Loigolactobacillus* were significantly enriched in WJ; *Lacticaseibacillus, Streptococcus* and *Kluyvera* were significantly enriched in LY15. The significantly enriched species in GY were *Lactiplantibacillus* sp., *Acetobacter indonesiensis*, *Levilactobacillus brevis*, *Pseudomonas* sp., *Lacticaseibacillus paracasei*, and *Pseudomonas putida*. Meanwhile, the significantly enriched species in WJ were *Bifidobacterium mongoliense, Loigolactobacillus coryniformis,* and *Lactiplantibacillus garii.* Finally, *Lacticaseibacillus manihotivorans* and *Lactobacillus* sp. were enriched in the LY15 sample. The differences in processing methods were one of the potential factors contributing to the variation in microbial community enrichment. LY15 and WJ samples have followed the same processing steps: soaking, grinding, precipitating, and sieving. Notably, during the soaking process, microorganisms such as *Lactobacillus plantarum* that were adhered to the surface of the mung beans were washed away [1]. In contrast, the GY sample was processed without soaking; the mung beans were directly ground and mixed with water in a specific ratio before fermentation, which preserved a higher number of *Lactobacillus plantarum* originally presented on the surface of the beans [2]. *Lactobacillus delbrueckii* was the dominant bacterial species in both WJ and LY15 samples and significantly enriched during fermentation. In fermented bean products, *Lactobacillus delbrueckii* effectively reduce the beany flavor and enhance the creaminess and flavor of plant-based yogurt [3]. Compared to *Lactobacillus plantarum*, *Lactobacillus delbrueckii* is less tolerant to acidic conditions. These previous studies have reported that when the pH drops below 4, *Lactobacillus delbrueckii* could enter a dormant state or even die, which could explain its absence in the GY sample, due to its pH being lower than 4 (Figure 1B) [6,37]. Integrated analysis of alpha and beta diversity revealed that LY15 and WJ samples demonstrated significantly higher microbial richness, while exhibiting greater similarity in microbial community composition compared to GY samples.

### 3.5. Functional Gene Category by Blasting to the EggNOG, KEGG, and CAZy Databases

**Figure 5:**
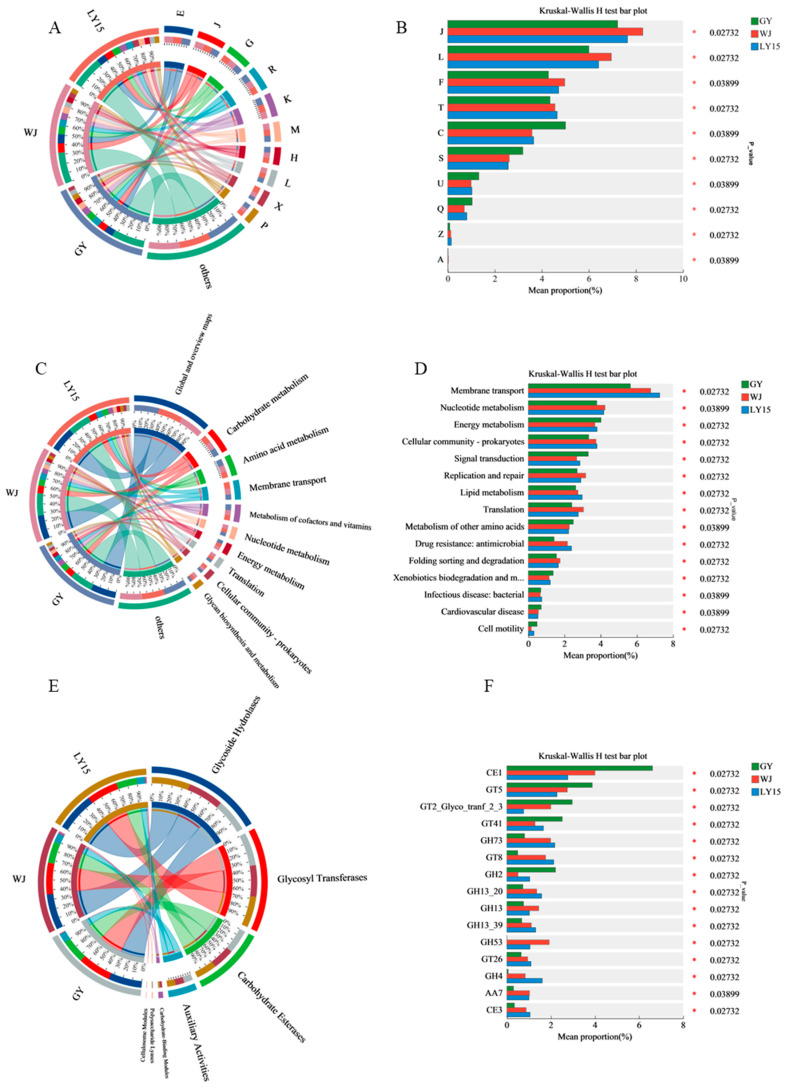
Functional gene annotation plots. Circos sample-function relationship plots are commonly used to show the distribution of functions present in different microbial samples. The bar graphs show the differences in the mean relative abundance of the same function between different groups and are annotated as to whether the differences are significant (*p*-value values and asterisks indicate significant differences). (**A**) COG annotated Circos plot. (**B**) COG annotated bar graph plot. (**C**) KEGG annotated Circos plot. (**D**) KEGG annotated bar graph plot. (**E**) CAZy annotated Circos plot. (**F**) CAZy annotated bar graph. * 0.01 < *p* ≤ 0.05.

Functional annotation of HMBS samples was conducted using the EggNOG, KEGG, and CAZy databases to compare metabolic functions. The Circos plot (Figure 5A,C,E) and bar graph (Figure 5B,D,F) highlighted functional distributions and relative abundances across samples. Gene sequences were aligned with EggNOG to identify orthologous groups (COG), revealing the top 10 functional genes (Figure 5A,B), including amino acid transport (9%), ribosomal structure (9%), carbohydrate metabolism (8%), and transcription (7%), with no significant differences in proportion across samples (7–9%). This indicates that all microbial communities are capable of meeting basic growth requirements.

According to the KEGG database (Figure 5C,D), WJ showed higher abundances of genes related to translation, ribosomal structure, nucleotide transport, and repair, indicating enhanced translation capacity and genomic stability, likely linked to *Bifidobacterium* and *Bifidobacterium mongoliense* [16,38]. GY exhibited higher levels of genes related to energy production, secretion, and vesicular transport, associated with *Lactobacillus plantarum*, suggesting enhanced energy metabolism and robust fermentation. LY15 displayed a balanced functional profile, indicating adaptability across diverse food systems. Statistical analysis confirmed significant functional differences among samples, with WJ showing strong potential for biosynthesis and probiotic applications (Figure 5D), GY excelling in fermentation, and LY15 offering versatile microbial functions.

Carbohydrate metabolism plays a vital role in HMBS fermentation that was ensured using EggNOG and KEGG annotations. Those results of CAZy revealed that glycoside hydrolases (GHs) were the most abundant functional genes (36%), playing vital roles in carbohydrate breakdown and fermentation (Figure 5E,F). GHs from genera like *Lactobacillus* and *Lactiplantibacillus* contributed to saccharification and lactic acid metabolism. Glycosyl transferases (GTs), the second most abundant genes, facilitated the production of monosaccharides and oligosaccharides, essential for organic acid and alcohol generation [39,40]. In GY, enzymes like CE1 (carbohydrate esterases 1) and GT5 (glycosyl transferases 5) were more abundant, indicating roles in cellulose digestion and prebiotic oligosaccharide production. Conversely, GH73 and GT8 enzymes, more abundant in WJ and LY15, aided in breaking down polysaccharides into monosaccharides and oligosaccharides, supporting further microbial utilization [41,42].

### 3.6. Analysis of the Differences in Carbohydrate and Amino Acid Metabolic Pathways

**Figure 6:**
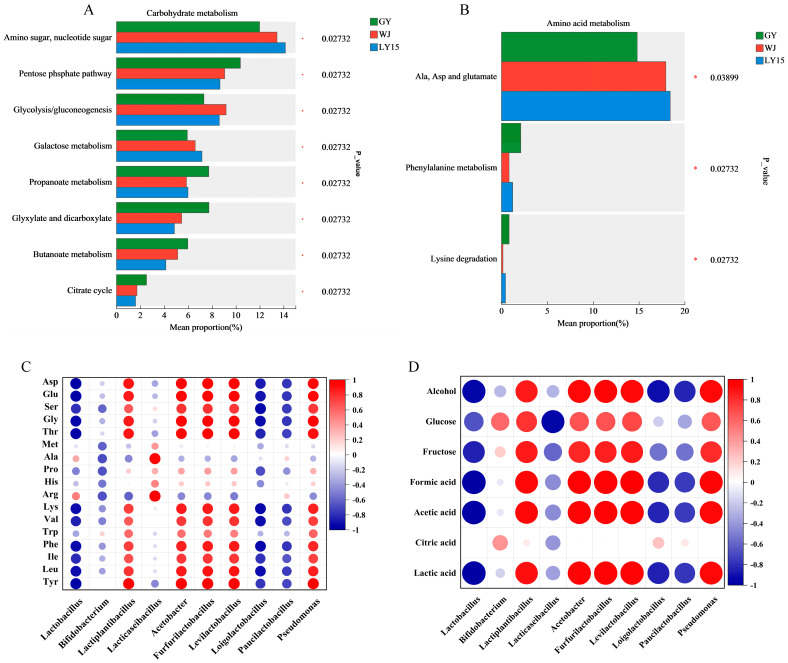
Differences in carbohydrate metabolism pathways across different samples (**A**) and differences in amino acid metabolism pathways (**B**). (**C**,**D**) represent the correlation between dominant bacterial genera and 17 free amino acids, as well as between even key metabolites, respectively. * means significant differences among samples (*p* < 0.05).

The protein and carbohydrates abundant in mung beans serve as substrates for microbial fermentation in HMBS. Degrading protein and starch could produce flavor compounds such as free amino acids, monosaccharides, and oligosaccharides [1,21]. Figure 6A,B illustrate the differential pathways of amino acid and carbohydrate metabolism in HMBS samples.

The relative abundance of functional genes related to amino sugar and nucleotide sugar metabolism (Ko00520) in the LY15 sample was higher than that in the GY and WJ samples, reaching nearly 14%. According to Supplementary—Figures S4 and S5, the high abundance of genes such as GDP-L-fucose synthase (1.1.1.271.), WlbA (1.1.1.335.), and UDP-GlcUA decarboxylase (1.1.1.305) was the primary reason for the enrichment of this metabolic pathway in the LY15 sample. In contrast, genes related to glycolysis/gluconeogenesis were significantly more abundant in the WJ sample compared to the GY and LY15 samples, which may be linked to the high abundance of *Lacticaseibacillus manihotivorans* in the WJ sample. Furthermore, genes involved the citrate cycle, propanoate metabolism, and glyoxylate and dicarboxylate metabolism were found in significantly higher abundance in the GY sample than in the WJ and LY15 samples. That was the potential factor that the energy metabolism observed in GY samples higher than WJ samples [43,44]. Alanine, aspartate, and glutamate metabolism, phenylalanine metabolism, and lysine degradation were three key differential pathways of free amino acids. Among these, the functional gene of alanine, aspartate, and glutamate metabolism was the highest abundance in the LY15 sample, followed by the WJ and GY samples (Figure 6B). The functional genes referred to this pathway were primarily responsible for the metabolism and conversion of Glu and Asp, which may explain the higher Glu content in the GY sample compared to WJ and LY15 [13]. Enzyme genes such as glutamate dehydrogenase (1.4.1.3), L-aspartate oxidase (1.4.3.16), and 4-aminobutyrate-pyruvate transaminase (2.6.1.96), although less abundant compared to other enzymes, have shown relatively higher abundance in the LY15 sample (Supplementary—Figures S2 and S3). This possibly contributed to the higher Glu content in the GY sample.

### 3.7. Correlation Analysis Between Dominant Genera and Vital Metabolites

The Pearson correlation was utilized to examine the potential relationships between dominant genera and metabolites, including free amino acids, monosaccharide, organic acids, and alcohol (Figure 6C,D). In the GY sample, *Lactiplantibacillus* was the dominant genus showing a strong positive correlation (r > 0.7) with amino acids such as Tyr, Leu, Ile, Gly, Asp, Glu, and Thr that was consistent with previous findings, suggesting *Lactiplantibacillus*’ involvement in glutathione metabolism [41]. In the GY sample, *Acetobacter*, *Levilactobacillus*, *Pseudomonas*, and *Furfurilactobacillus* took advantage of the proportion and exhibited positive correlations (r > 0.7) with amino acids like Glu, Asp, Ser, Tyr, Lys, and Leu, indicating potential synergistic roles in amino acid metabolism [7,45]. On the contrary, dominant genera in the WJ and LY15 samples, including *Lactobacillus*, *Bifidobacterium*, *Loigolactobacillus*, and *Paucilactobacillus*, showed negative correlations (r < 0.6) with most amino acids, particularly Asp, Glu, Phe, and Lys. This may reflect that *Lactobacillus* possessed superior gene for amino acid utilization, enabling it to convert and utilize free amino acids more efficiently [46,47]. Figure 6D illustrate the correlations between dominant genera and metabolites, suggesting complex metabolic interactions. In the GY sample, *Lactiplantibacillus*, *Acetobacter*, *Furfurilactobacillus*, *Pseudomonas*, and *Levilactobacillus* were positively correlated (r > 0.7) with organic acids (lactic, acetic, and formic acids) and ethanol, indicating high metabolic efficiency in producing these compounds and tolerance to the harsh environments of low pH [9,39]. This may be the reason for the lower pH of the GY sample. *Lactobacillus* in WJ and LY15 samples displayed negative correlations with lactic acid, acetic acid, and glucose (r < 0.7), likely due to lactic acid accumulation inhibiting further *Lactobacillus* growth, while other genera like *Acetobacter* may metabolize lactic acid into acetic acid.

These interactions suggest competitive or cooperative utilization of metabolites in co-fermentation systems, reflecting the principles of “metabolic networks” and “cross-feeding” in microbial ecology. The partitioning of metabolites in microbial communities optimizes resource utilization and balances inhibitory effects, maintaining equilibrium. Such dynamics are crucial for optimizing fermentation processes in HMBS, where the accumulation of metabolites like lactic acid can inhibit certain species while promoting others to utilize secondary substrates [48].

## 4. Conclusions

In summary, this study investigated the concentrations of 17 free amino acids and seven important metabolites in three types of HMBS. It was found that the umami amino acids, Asp and Glu, were the most abundant free amino acids with a TVA greater than 1. Among the organic acids, lactic acid demonstrated the highest concentration, with levels significantly exceeding those of other metabolites in this group. Metagenomic analysis revealed the microbial diversity in the HMBS samples, including the member composition of the microbial communities and their functional genes. The dominant genera in the WJ and LY15 samples were *Lactobacillus* and *Bifidobacterium*. However, *Lactiplantibacillus* and *Acetobacter* were the predominant genera in the GY sample. The functional genes of the microbial communities were primarily associated with carbohydrate and amino acid metabolism. Both *Lactiplantibacillus* and *Acetobacter* showed significant positive correlations with most amino acids and organic acids, whereas *Lactobacillus* and *Loigolactobacillus* kept significant negative correlations with these metabolites. In the next phase of the research, we will integrate metabolomic and metagenomic approaches to conduct a comprehensive analysis of metabolic pathways within HMBS. Additionally, the functional gene expression identified in these pathways will be further validated using qPCR assays in follow-up experiments.

In conclusion, this study identifies the dominant microbial genera and characterizes key metabolites in HMBS samples, establishing a scientific foundation for its future industrial-scale production and quality standardization.

## Figures and Tables

**Table 1:** The contents, VIP, and TVA of free amino acid in Henan mung bean sour. VIP means variable importance in the projection. T means taste threshold. Within the same row, values marked with different superscript letter (a–c) indicate a difference at *p* < 0.05.

Amino Acid		GY	WJ	LY15	GY	WJ	LY15	
					TVA			VIP
Umamiiii	T							
Asp	10	66 ± 16.46 ^a^	3.66 ± 0.57 ^b^	10.3333 ± 0.57 ^b^	6.6	0.366	1.033	0.91
Glu	3	109 ± 25.15 ^a^	56.66 ± 2.08 ^b^	70 ± 1.01 ^b^	36.33	18.88	23.33	0.92
TUAA		175	59	80				
Sweet								
Ser	15	18.33 ± 1.15 ^c^	8.33 ± 1.15 ^b^	14.33 ± 0.57 ^a^	1.22	0.55	0.96	1.02
Gly	13	29 ± 4 ^c^	5.66 ± 0.57 ^b^	12.66 ± 2.08 ^a^	2.23	0.43	0.92	1.03
Thr	26	21 ± 3.46 ^a^	4 ± 1.10 ^b^	5.66 ± 0.57 ^b^	0.80	0.15	0.21	1.01
Met	6	11.33 ± 3.21	9.66 ± 0.57	12 ± 1.12	3.66	3.22	4	0.90
Ala	30	31 ± 1.03 ^b^	28 ± 2.64 ^b^	43.66 ± 0.57 ^a^	5.16	4.66	7.16	1.18
Pro	50	7.66 ± 1.15 ^a^	5.66 ± 0.57 ^b^	7.33 ± 0.57 ^a^	0.25	0.18	0.24	0.59
TSAA		117	59	93				
Bitter								
His	90	12.33 ± 0.57 ^a^	10.66 ± 1.15 ^b^	12.66 ± 0.57 ^a^	6.16	5.33	6.33	0.83
Arg	5	0.5 ± 0.04 ^b^	0.51 ± 0.02 ^b^	14.33 ± 2.08 ^a^	0.1	0.102	2.86	1.28
Lys	5	20.66 ± 1.52 ^c^	8.33 ± 0.05 ^b^	13 ± 1.11 ^a^	4.132	1.66	2.6	0.99
Val	4	30 ± 3.60 ^a^	9 ± 7.81 ^b^	21 ± 1.00 ^a^	7.56	2.25	5.25	1.04
Trp	9	1.33 ± 0.15	0.66 ± 0.57	0.66 ± 0.05	0.14	0.07	0.07	
Phe	9	22 ± 1.02 ^a^	13 ± 1 ^c^	16 ± 1.13 ^b^	2.44	1.44	1.77	1.00
Ile	9	13 ± 2.64 ^a^	7.67 ± 0.57 ^b^	9.33 ± 0.57 ^b^	1.44	0.85	1.03	0.96
Leu	19	34 ± 2.64 ^a^	16.66 ± 0.57 ^c^	22.33 ± 2.08 ^b^	1.78	0.87	1.17	1.04
TBAA		132	63	107				
Astringent								
Tyr	9.1	13.66 ± 2.51 ^a^	1.33 ± 0.57 ^b^	0.66 ± 0.04 ^b^	1.50	0.14	0.07	1.03
TAAA		13	1	0.66				

**Table 2:** Proportion of the top ten most abundant genera across different samples at the genus level. Different letters indicate a significant difference among samples. Nd means not detected.

Genus	GY (%)	LY15 (%)	WJ (%)
*Lactobacillus*	0.24 ± 0.03b	41.23 ± 0.56a	40.48 ± 2.32a
*Bifidobacterium*	16.38 ± 5.78b	7.75 ± 2.11c	28.98 ± 9.72a
*Lactiplantibacillus*	34.40 ± 9.79a	3.78 ± 0.48c	10.24 ± 0.45b
*Lacticaseibacillus*	3.25 ± 0.41b	20.58 ± 2.49a	2.27 ± 0.09b
*Acetobacter*	15.27 ± 4.37a	Nd	Nd
*Furfurilactobacillus*	10.29 ± 2.43a	Nd	Nd
*Levilactobacillus*	8.06 ± 1.41a	0.64 ± 0.03c	1.00 ± 0.29b
*Loigolactobacillus*	0.49 ± 0.07c	3.03 ± 0.21b	5.50 ± 1.50a
*Paucilactobacillus*	Nd	3.80 ± 0.82	4.77 ± 3.52
*Pseudomonas*	7.40 ± 2.74	Nd	Nd

## Data Availability

The original contributions presented in this study are included in the article/Supplementary Material. Further inquiries can be directed to the corresponding author.

## Supplementary Materials

The following supporting information can be downloaded at: https://www.mdpi.com/article/10.3390/microorganisms13040845/s1, Figure S1: Abundance difference of three samples at phylum level; Figure S2: Alanine, aspartate and glutamate metabolism; Figure S3: The relative abundance changes of vital enzymes in alanine, aspartate and glutamate metabolism and glutamate metabolism; Figure S4: Amino sugar and nucleotide metabolism; Figure S5: The vital enzymes changes in amino sugar and nucleotide metabolism; Table S1: Abundance difference of three samples at species level.

## References

[1] Abdeldaiem A.M., Ali A.H., Shah N., Ayyash M., Mousa A.H. (2023). Physicochemical analysis, rheological properties, and sensory evaluation of yogurt drink supplemented with roasted barley powder. LWT.

[2] An T., Chen M., Zu Z., Chen Q., Lu H., Yue P., Gao X. (2021). Untargeted and targeted metabolomics reveal changes in the chemical constituents of instant dark tea during liquid-state fermentation by Eurotium cristatum. Food Res. Int..

[3] Bao T., Deng S., Yu K., Li W., Dong A. (2020). Metagenomic insights into seasonal variations in the soil microbial community and function in a Larix gmelinii forest of Mohe, China. J. For. Res..

[4] Behera B.C., Mishra R., Mohapatra S. (2021). Microbial citric acid: Production, properties, application, and future perspectives. Food Front..

[5] Chen J.-B., Li G., Chen X., Liao L.-H., He Y.-Q., Ye F., Chen G.-X. (2025). Nutritional value and antioxidant activity of Artemisia princeps, an edible plant frequently used in folk food in the Xiangxi region. Food Med. Homol..

[6] Chen Y.-W., Yu Y.-H. (2023). Differential effects of Bacillus subtilis– and Bacillus licheniformis–fermented products on growth performance, intestinal morphology, intestinal antioxidant and barrier function gene expression, cecal microbiota community, and microbial carbohydrate-active enzyme composition in broilers. Poult. Sci..

[7] Chen Z., Liang N., Zhang H., Li H., Guo J., Zhang Y., Chen Y., Wang Y., Shi N. (2024). Resistant starch and the gut microbiome: Exploring beneficial interactions and dietary impacts. Food Chem. X.

[8] Cui J., Xia P., Zhang L., Hu Y., Xie Q., Xiang H. (2020). A novel fermented soybean, inoculated with selected Bacillus, Lactobacillus and Hansenula strains, showed strong antioxidant and anti-fatigue potential activity. Food Chem..

[9] De Vuyst L., Leroy F. (2020). Functional role of yeasts, lactic acid bacteria and acetic acid bacteria in cocoa fermentation processes. FEMS Microbiol. Rev..

[10] Derrien M., Turroni F., Ventura M., van Sinderen D. (2022). Insights into endogenous Bifidobacterium species in the human gut microbiota during adulthood. Trends Microbiol..

[11] Du X., Xu Y., Jiang Z., Zhu Y., Li Z., Ni H., Chen F. (2021). Removal of the fishy malodor from Bangia fusco-purpurea via fermentation of *Saccharomyces cerevisiae*, *Acetobacter pasteurianus*, and *Lactobacillus plantarum*. J. Food Biochem..

[12] Echegaray N., Yilmaz B., Sharma H., Kumar M., Pateiro M., Ozogul F., Lorenzo J.M. (2023). A novel approach to Lactiplantibacillus plantarum: From probiotic properties to the omics insights. Microbiol. Res..

[13] Gao P., Zhang Z., Jiang Q., Hu X., Zhang X., Yu P., Yang F., Liu S., Xia W. (2025). Metabolomics ravels flavor compound formation and metabolite transformation in rapid fermentation of salt-free fish sauce from catfish frames induced by mixed microbial cultures. Food Chem..

[14] Gao R., Peng P., Yu L., Wan B., Liang X., Liu P., Liao W., Miao L. (2024). Metagenomic analysis reveals the correlations between microbial communities and flavor compounds during the brewing of traditional Fangxian huangjiu. Food Biosci..

[15] Han D., Yang Y., Guo Z., Dai S., Jiang M., Zhu Y., Wang Y., Yu Z., Wang K., Rong C. (2024). A Review on the Interaction of Acetic Acid Bacteria and Microbes in Food Fermentation: A Microbial Ecology Perspective. Foods.

[16] Hao H., Yan R., Miao Z., Wang B., Sun J., Sun B. (2022). Volatile organic compounds mediated endogenous microbial interactions in Chinese baijiu fermentation. Int. J. Food Microbiol..

[17] Hong J.H., Jin Y.H., Pawluk A.M., Mah J.-H. (2024). Metagenomic and culturomic analyses of bacterial species contributing to tyramine formation in Cheonggukjang. LWT.

[18] Hu Y., Zhang L., Wen R., Chen Q., Kong B. (2020). Role of lactic acid bacteria in flavor development in traditional Chinese fermented foods: A review. Crit. Rev. Food Sci. Nutr..

[19] Jung S., Lee J.-H. (2020). Characterization of transcriptional response of Lactobacillus plantarum under acidic conditions provides insight into bacterial adaptation in fermentative environments. Sci. Rep..

[20] Kim S.Y., Hyeonbin O., Lee P., Kim Y.-S. (2020). The quality characteristics, antioxidant activity, and sensory evaluation of reduced-fat yogurt and nonfat yogurt supplemented with basil seed gum as a fat substitute. J. Dairy Sci..

[21] Kircher B., Woltemate S., Gutzki F., Schlüter D., Geffers R., Bähre H., Vital M. (2022). Predicting butyrate- and propionate-forming bacteria of gut microbiota from sequencing data. Gut Microbes..

[22] (2016). Determination of Amino Acids in Food Safety National Standards.

[23] Kuang X., Su H., Li W., Lin L., Lin W., Luo L. (2022). Effects of microbial community structure and its co-occurrence on the dynamic changes of physicochemical properties and free amino acids in the Cantonese soy sauce fermentation process. Food Res. Int..

[24] Lee H.S., Song M.W., Kim K.-T., Hong W.-S., Paik H.-D. (2021). Antioxidant Effect and Sensory Evaluation of Yogurt Supplemented with Hydroponic Ginseng Root Extract. Foods.

[25] Lee Y., Oh J., Jeong Y.-S. (2015). Lactobacillus plantarum-mediated conversion of flavonoid glycosides into flavonols, quercetin, and kaempferol in Cudrania tricuspidata leaves. Food Sci. Biotechnol..

[26] Li H., Li B., Gao L., Ge R., Cui X., Zhou J., Li Z. (2023). Gamma-aminobutyric acid (GABA) promotes characteristics of Levilactobacillus sp. LB-2. LWT.

[27] Li S., Feng X., Hao X., Zhu Y., Zou L., Chen X., Yao Y. (2023). A comprehensive review of mung bean proteins: Extraction, characterization, biological potential, techno-functional properties, modifications, and applications. Compr. Rev. Food Sci. Food Saf..

[28] Li W., Shen X., Wang J., Sun X., Yuan Q. (2021). Engineering microorganisms for the biosynthesis of dicarboxylic acids. Biotechnol. Adv..

[29] Li X., Wu Y., Shu L., Zhao L., Cao L., Li X., Tie S., Tian P., Gu S. (2024). Unravelling the correlations among the microbial community, physicochemical properties, and volatile compounds of traditional mung bean sour liquid. LWT.

[30] Liu M., Deng N., Hou X., Zhang B., Li H., Wang J. (2024). Characterisation of flavour profiles and microbial communities of fermented peppers with different fermentation years by combining flavouromics and metagenomics. Food Chem..

[31] Nyanzi R., Jooste P.J., Buys E.M. (2021). Invited review: Probiotic yogurt quality criteria, regulatory framework, clinical evidence, and analytical aspects. J. Dairy Sci..

[32] Peng X., Liao Y., Ren K., Liu Y., Wang M., Yu A., Tian T., Liao P., Huang Z., Wang H. (2022). Fermentation performance, nutrient composition, and flavor volatiles in soy milk after mixed culture fermentation. Process Biochem..

[33] Que Z., Jin Y., Huang J., Zhou R., Wu C. (2023). Flavor compounds of traditional fermented bean condiments: Classes, synthesis, and factors involved in flavor formation. Trends Food Sci. Technol..

[34] Ricke S.C., Dittoe D.K., Richardson K.E. (2020). Formic Acid as an Antimicrobial for Poultry Production: A Review. Front. Vet. Sci..

[35] Shangpliang H.N.J., Tamang J.P. (2023). Metagenome-assembled genomes for biomarkers of bio-functionalities in Laal dahi, an Indian ethnic fermented milk product. Int. J. Food Microbiol..

[36] Tarahi M., Abdolalizadeh L., Hedayati S. (2024). Mung bean protein isolate: Extraction, structure, physicochemical properties, modifications, and food applications. Food Chem..

[37] Tokatli DemİRok N., Alpaslan M., YikmiŞ S. (2023). Some lactobacillus, leuconostoc and acetobacter strains in traditional turkish yoghurt, cheese, kefir samples as a probiotic candidate. Int. J. Agric. Environ. Food Sci..

[38] Wang X., Wang X., Lou H., Li Y., khashaba R., Zhao R. (2024). Understanding the correlation between formation of flavor compounds and dominant bacteria during Luoyang mung bean sour fermentation. Food Biosci..

[39] Wang Y., Wang Y., Qiu S., Wang B., Zeng H. (2024). Metagenomic and flavoromic profiling reveals the correlation between the microorganisms and volatile flavor compounds in Monascus-fermented cheese. Food Res. Int..

[40] Xia A., Yang Y., Guo M., Lee Y.-K., Yang B., Liu X., Zhao J., Zhang H., Chen W. (2023). Unveiling of the key pathway in flavor formation in fermented milk of Lactococcus lactis subsp. lactis via genomics and metabolomics. Food Biosci..

[41] Xia F., Hu S., Zheng X., Wang M.W., Zhang C.C., Wu Z.N., Sun Y.J. (2022). New insights into metabolomics profile generation in fermented tea: The relevance of bacteria and metabolites in Fuzhuan brick tea. J. Sci. Food Agric..

[42] Xu H., Chen Y., Ding S., Qin Y., Jiang L., Zhou H., Deng F., Wang R. (2021). Changes in texture qualities and pectin characteristics of fermented minced pepper during natural and inoculated fermentation process. Int. J. Food Sci. Technol..

[43] Xu J., Peng S., Xiong Y., Zheng Z., Liu M., Xu J., Chen W., Liu M., Kong J., Wang C. (2024). A review on fermented vegetables: Microbial community and potential upgrading strategy via inoculated fermentation. Compr. Rev. Food Sci. Food Saf..

[44] Xu Y., Yu Y., Tian Y., Su Y., Li X., Zhang Z., Zhu H., Han J., Zhang H., Liu L. (2018). Analysis of Beijing Douzhir Microbiota by High-Throughput Sequencing and Isolation of Acidogenic, Starch-Flocculating Strains. Front. Microbiol..

[45] Yu M., Li Z., Rong T., Tian Z., Deng D., Lu H., Zhang R., Ma X. (2022). Integrated metagenomics-metabolomics analysis reveals the cecal microbial composition, function, and metabolites of pigs fed diets with different starch sources. Food Res. Int..

[46] Yu Y., Xu Y., Li L., Chen S., An K., Yu Y., Xu Z.-L. (2023). Isolation of lactic acid bacteria from Chinese pickle and evaluation of fermentation characteristics. LWT.

[47] Zhao Y.-L., Tang R., Liu S., Han S.-T., Feng J., Chi K.-X., Yang G., Hou X.-Y., Fang Y.-W. (2025). Effects of urolithin A-producing Streptococcus thermophilus FUA329 fermentation on the composition and antioxidant bioactivities of black tea. Food Med. Homol..

[48] Glen D., Shraddha S., Daniel P., Ghada Y., Silvio W., Christian K. (2018). Ecology and evolution of metabolic cross-feeding interactions in bacteria. Nat. Prod. Rep..

