# Supplementary material for: Revealing the Characteristics and Correlations Among Microbial Communities, Functional Genes, and Vital Metabolites Through Metagenomics in Henan Mung Bean Sour
Source: Microorganisms. 2025 Apr 7;13(4):845. doi: 10.3390/microorganisms13040845 (PMC12029783; doi:10.3390/microorganisms13040845)

Supplementary Figure S1

Abundance difference of three samples at phylum level

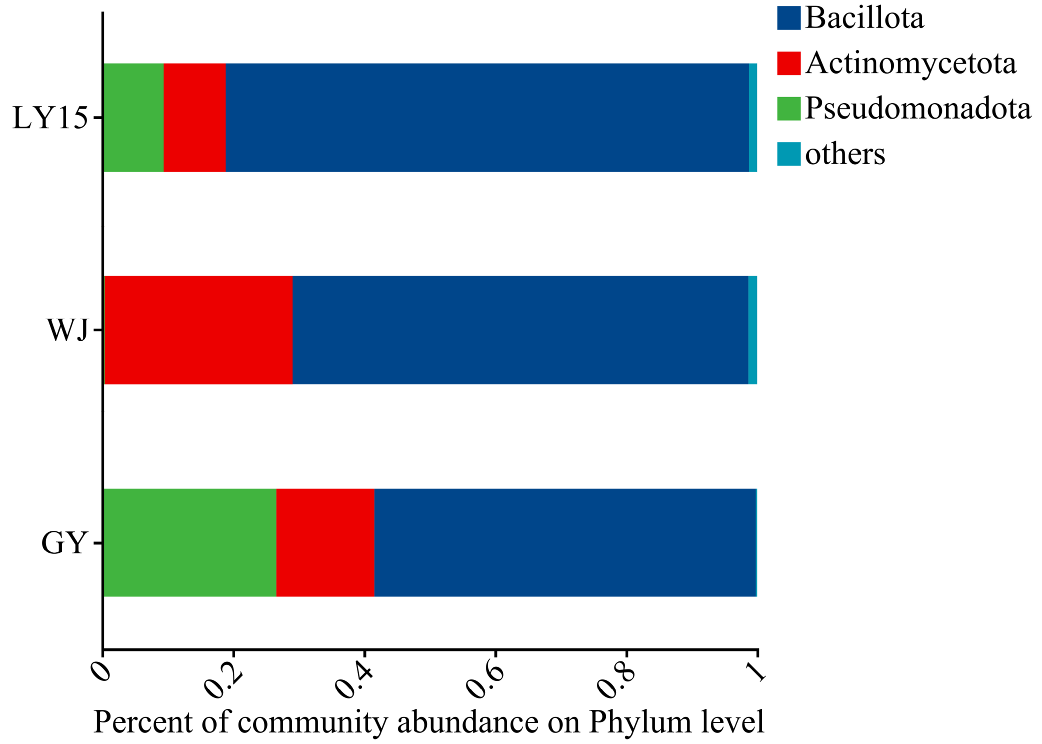

Supplementary Table S1: Abundance difference of three samples at species level

Species	GY	LY15	WJ
Lactobacillus delbrueckii	Nd	31.45±0.49	31.08±1.73
Bifidobacterium mongoliense	14.76±5.40b	6.85±1.93c	25.50±9.03a
Lactiplantibacillus plantarum	21.31±5.89a	1.51±0.15b	1.64±0.50b
Lactobacillus sp.	0.19±0.02b	8.94±0.12a	8.51±0.44a
Lacticaseibacillus manihotivorans	0.02±0.005c	14.94±1.83a	0.83±0.03b
Lactiplantibacillus sp.	12.47±3.63a	0.43±0.03b	0.57±0.08b
Acetobacter indonesiensis	12.41±3.65a	Nd	Nd
Levilactobacillus brevis	7.96±1.38a	0.34±0.05b	0.55±0.27b
Paucilactobacillus nenjiangensis	Nd	3.53±0.78b	4.46±3.36a
Loigolactobacillus coryniformis	0.47±0.06c	2.53±0.19b	3.08±0.73a
Lacticaseibacillus paracasei	3.01±0.40a	1.23±0.12b	0.79±0.09c
Pseudomonas sp	4.20±1.56	Nd	Nd
Furfurilactobacillus_sp.	4.06±1.00	Nd	Nd
Lactiplantibacillus garii	Nd	Nd	3.93±0.69
Furfurilactobacillus milii	3.30±0.72	Nd	Nd
Lactiplantibacillus mudanjiangensis	Nd	1.54±0.26	1.15±0.16
Loigolactobacillus bifermentans	Nd	0.41±0.05b	2.26±0.72a
Latilactobacillus curvatus	Nd	1.19±0.21	1.35±0.41
Furfurilactobacillus rossiae	2.62±0.70	Nd	Nd

Supplementary Figure S2: Alanine, aspartate and glutamate metabolism

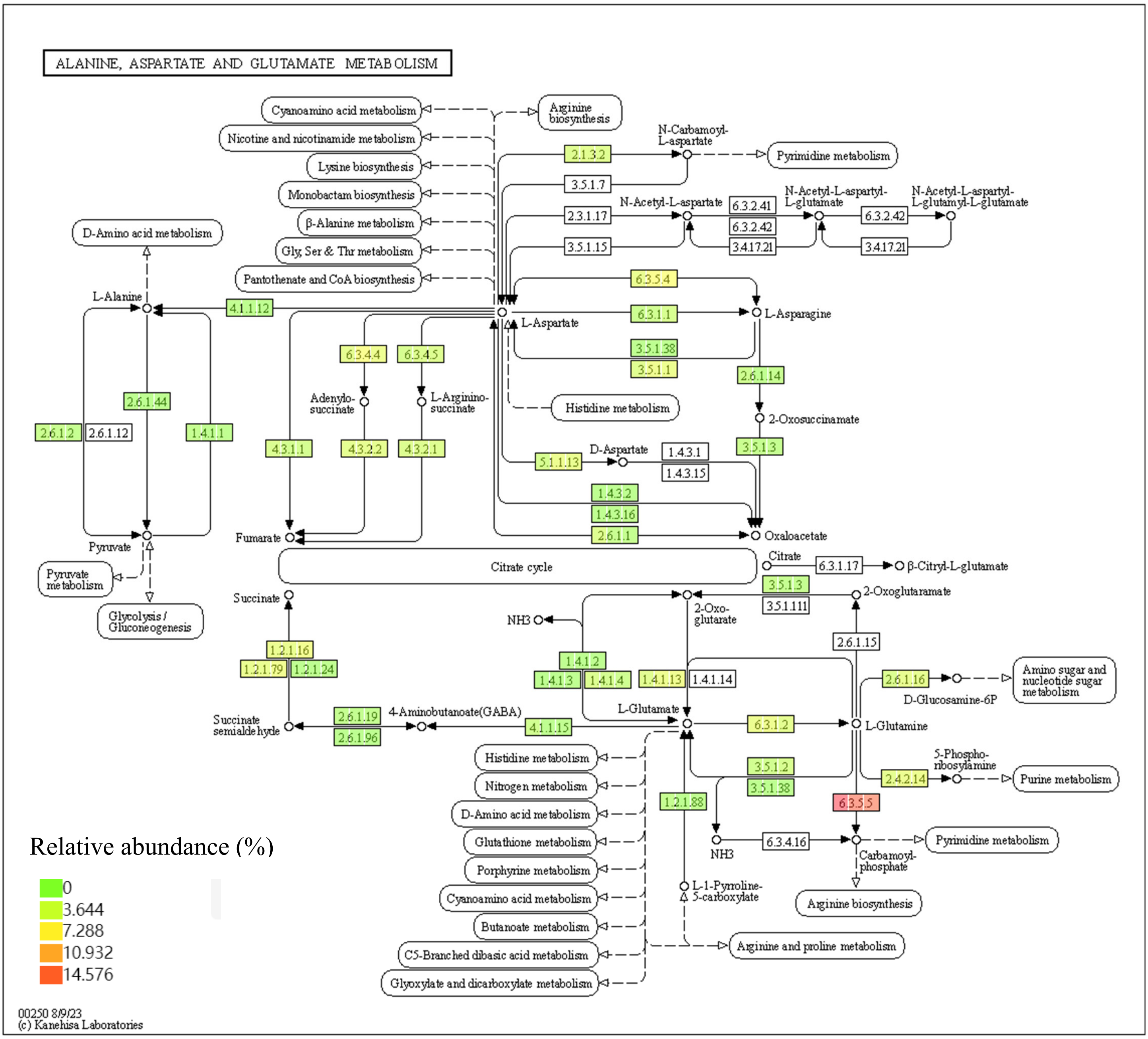

Each box with a fill Each box with a fill color represents one or a group of samples, and the depth of the color represents the abundance of the enzyme in different samples or groups

Supplementary Figure S3: The relative abundance changes of vital enzymes in alanine, aspartate and glutamate metabolism and glutamate metabolism

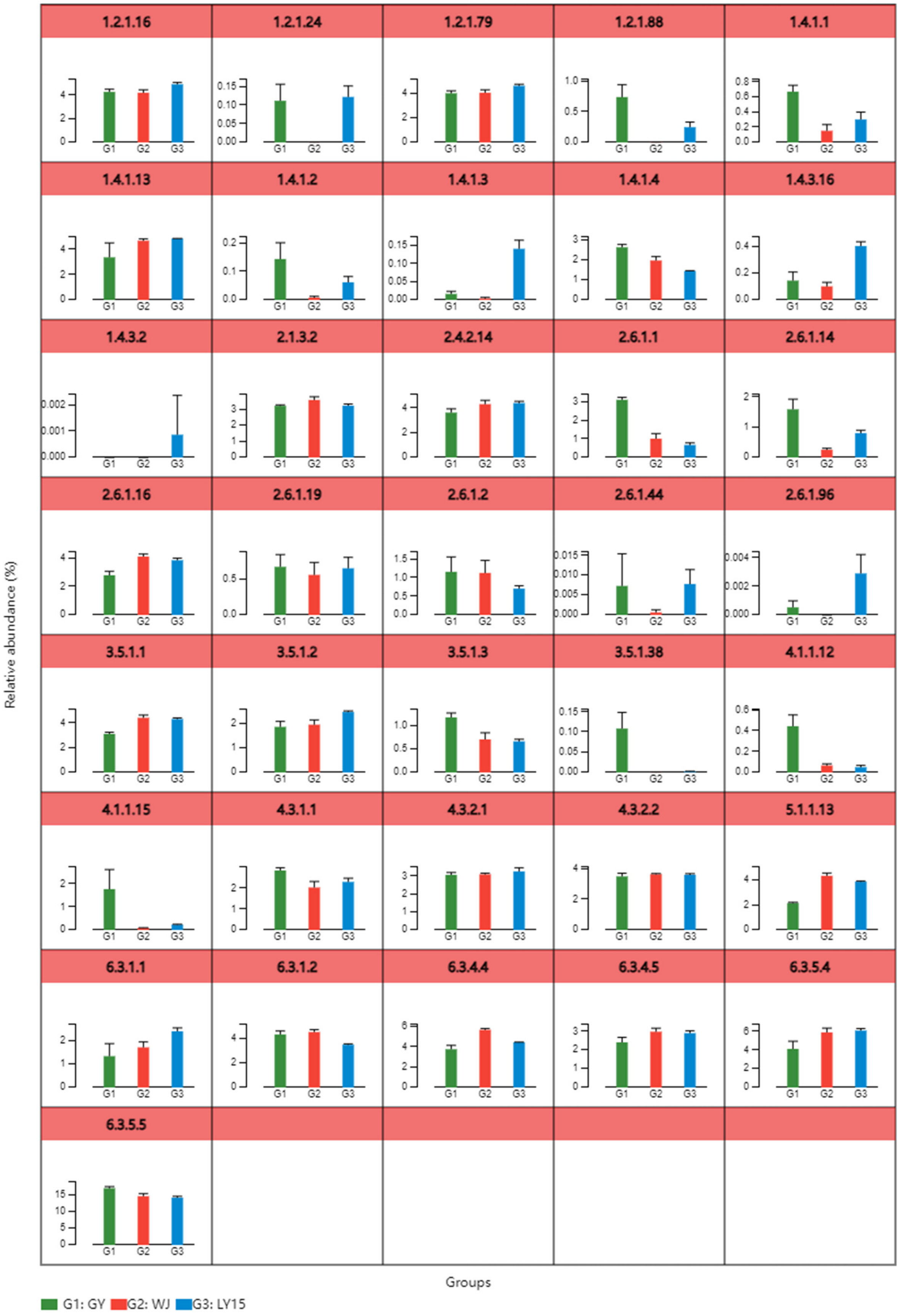

Supplementary Figure S4: Amino sugar and nucleotide metabolism

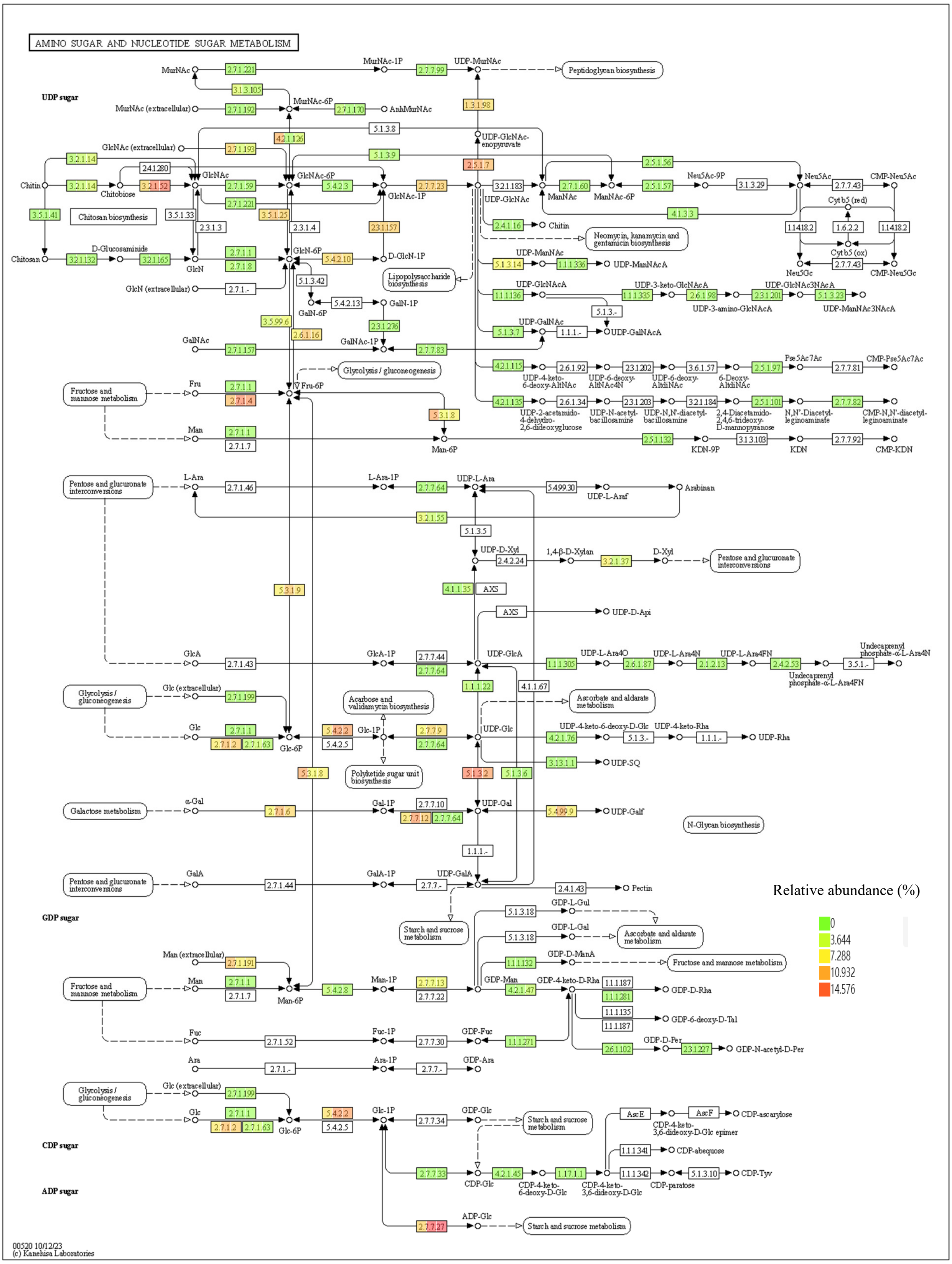

Each box with a fill Each box with a fill color represents one or a group of samples, and the depth of the color represents the abundance of the enzyme in different samples or groups

Supplementary Figure S5: The vital enzymes changes in amino sugar and nucleotide metabolism

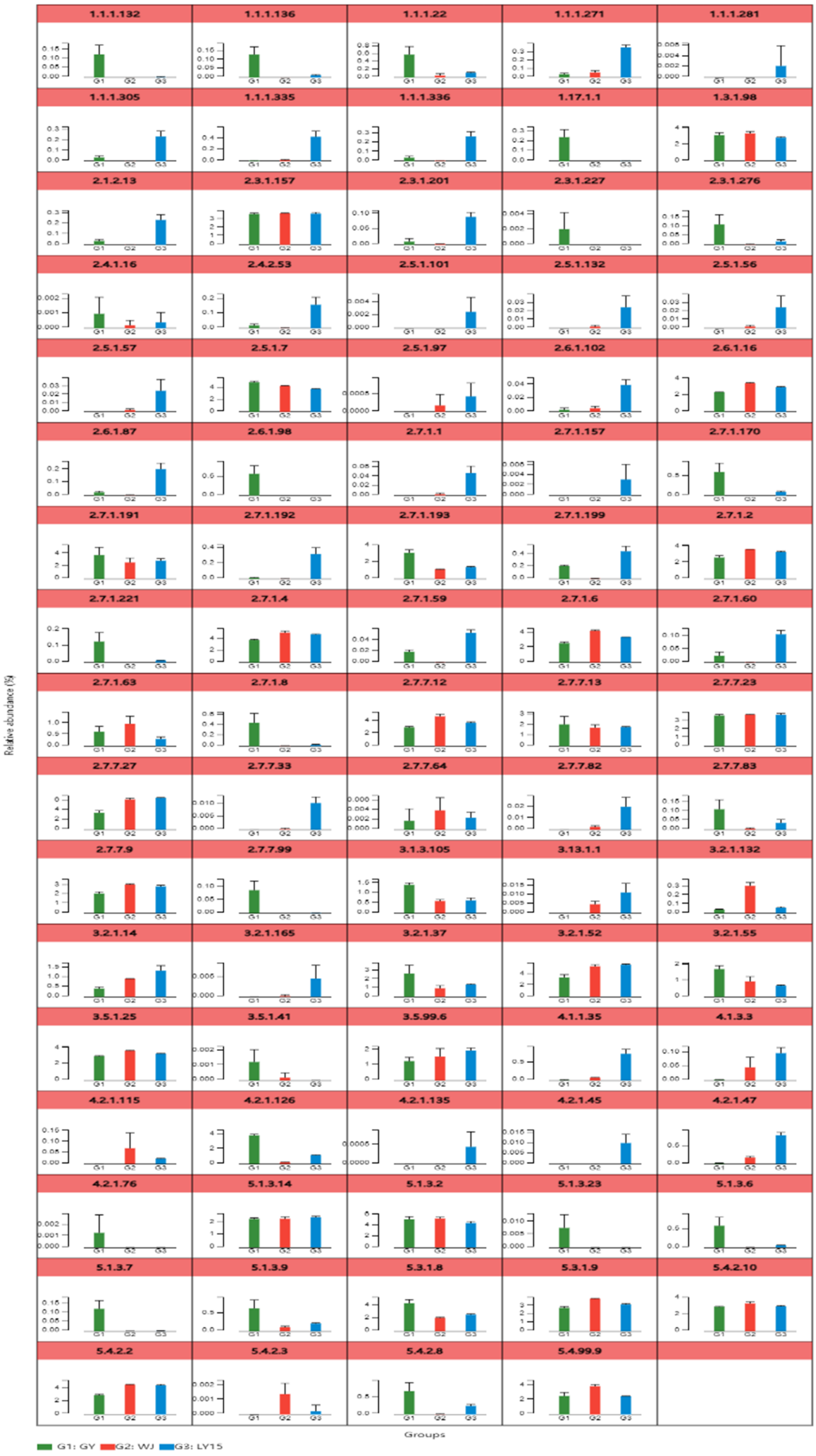

Supplement: Supplementary file 1 [file microorganisms-13-00845-s001.zip › microorganisms-3545803-supplementary.pdf]
